# First-line treatment for patients with advanced non-small cell lung carcinoma and high PD-L1 expression: pembrolizumab or pembrolizumab plus chemotherapy

**DOI:** 10.1186/s40425-019-0600-6

**Published:** 2019-05-03

**Authors:** Yixin Zhou, Zuan Lin, Xuanye Zhang, Chen Chen, Hongyun Zhao, Shaodong Hong, Li Zhang

**Affiliations:** 1 State Key Laboratory of Oncology in South China, Guangzhou, China; 2 Collaborative Innovation Center for Cancer Medicine, Guangzhou, China; 3 Department of VIP region, Sun Yat-sen University Cancer Center, Guangzhou, China; 4 Department of Clinical Research, Sun Yat-sen University Cancer Center, Guangzhou, China; 5 Department of Medical Oncology, Sun Yat-sen University Cancer Center, 651 Dongfeng East Road, Guangzhou, 510060 China; 6 Department of Radiotherapy, Sun Yat-sen University Cancer Center, Guangzhou, China

**Keywords:** Non-small cell lung cancer, Programmed cell death-ligand 1, Pembrolizumab, Chemotherapy, First-line

## Abstract

Pembrolizumab monotherapy has become the preferred treatment for patients with advanced non-small cell lung carcinoma (NSCLC) and a programmed cell death-ligand 1 (PD-L1) tumor proportion score (TPS) of at least 50%. However, little is known about the value of adding chemotherapy to pembrolizumab in this setting. Therefore, we performed an indirect comparison for pembrolizumab plus chemotherapy versus pembrolizumab, using the frequentist methods. The primary outcomes were overall survival (OS), progression-free survival (PFS) and objective response rate (ORR). Data were retrieved from randomized trials comparing pembrolizumab plus chemotherapy or pembrolizumab monotherapy against chemotherapy. Five trials involving 1289 patients were included. Direct meta-analysis showed that both pembrolizumab plus chemotherapy (ORR: relative risk (RR) 2.16; PFS: hazard ratio (HR) 0.36; OS: HR 0.51) and pembrolizumab alone (ORR: RR 1.33; PFS: HR, 0.65; OS: HR 0.67) improved clinical outcomes compared with chemotherapy. Indirect comparison showed that pembrolizumab plus chemotherapy was superior to pembrolizumab alone, in terms of ORR (RR 1.62, 1.18–2.23) and PFS (HR 0.55, 0.32–0.97). A trend towards improved OS was also observed (HR 0.76, 0.51–1.14). In conclusion, the addition of chemotherapy to pembrolizumab further improves the outcomes of patients with advanced NSCLC and a PD-L1 TPS of at least 50%.

**Electronic supplementary material:**

The online version of this article (10.1186/s40425-019-0600-6) contains supplementary material, which is available to authorized users.

## Introduction

With recent advance of immune checkpoint inhibitor treatment that blocks the PD-1 (programmed cell death 1) and PD-L1 (programmed cell death-ligand 1) pathway, pembrolizumab monotherapy has replaced platinum-doublet chemotherapy as first-line treatment in patients with advanced non-small cell lung carcinoma (NSCLC) and a PD-L1 tumor proportion score (TPS) of 50% or more [1]. Among patients with unselected PD-L1 expression, pembrolizumab plus chemotherapy is superior to chemotherapy alone [2]. However, whether combination of pembrolizumab and chemotherapy could further improve the clinical outcomes compared with pembrolizumab alone remains an urgent controversy due to the lack of head-to-head comparison.

We evaluated the efficacy of pembrolizumab (pem) plus chemotherapy (chemo) versus pembrolizumab alone for the first-line treatment of patients with advanced NSCLC and a PD-L1 TPS of ≥50% using indirect comparison meta-analysis.

## Methods

### Study eligibility

We identified eligible randomized controlled trials that compared pembrolizumab plus chemotherapy or pembrolizumab alone with chemotherapy for first-line treatment of advanced NSCLC from Pubmed, Embase and the Cochrane Central Register, with the search terms including pembrolizumab, non–small cell lung cancer, and randomized controlled trial (Additional file 1: Supplemental Methods). The abstracts from major conference proceedings of the American Society of Clinical Oncology (ASCO), the European Society of Medical Oncology (ESMO), the American Association for Cancer Research (AACR), and the World Conference on Lung Cancer (WCLC) were also reviewed. Studies were restricted to English language published or presented before November 1, 2018.

### Data extraction

Data were extracted with a predefined information sheet. The primary outcomes for this study were overall survival (OS), progression-free survival (PFS) and objective response rate (ORR). We extracted the hazard ratios (HRs) and their 95% confidence intervals (CIs) for OS and PFS, and dichotomous data for ORR. Other items included acronym of the trial, number of patients enrolled, and clinicopathological characteristics of the patients.

### Data analyses

Direct comparisons were performed for arm A (pembrolizumab plus chemotherapy) versus arm C (chemotherapy), and arm B (pembrolizumab) versus arm C (chemotherapy), respectively. The pooled estimates for PFS and OS were presented with HRs, 95% CIs and *P* values calculated using the inverse-variance-weighted method, while the measures for dichotomous data (ORR) were pooled with the relative risks (RRs), 95% CIs and *P* values using the Mantel Haenszel method. A fixed-effect or random-effect model was adopted depending on between-study heterogeneity.

Indirect comparison was performed for arm A versus arm B, linked by arm C. The adjusted indirect comparison was calculated using the frequentist methods with the following formulas [3]: log HR_AB_ = log HR_AC_-log HR_BC_, and its standard error (SE) for the log HR was $SE\ \left(\mathit{\log} HR\mathrm{AB}\right)=\sqrt{SE{\left(\mathit{\log} HR\mathrm{AC}\right)}^2+ SE{\left(\mathit{\log} HR\mathrm{BC}\right)}^2}$. RR was calculated similarly as the above formulas. HR < 1 or RR > 1 indicates that pembrolizumab plus chemotherapy is superior to pembrolizumab alone, vice versa.

All statistical analyses were conducted using SAS statistical software (version 15.0, SAS Institute Inc). Statistical significance was defined as a 2-sided *P* < .05.

## Results

A total of five trials involving 1289 patients were included (trial selection process shown in Additional file 1: Figure S1) [1, 4–7]. The assessment of risk of bias is presented in Additional file 1: Table S1.

The main characteristics and outcomes of the included trials are summarized in Table 1. Three trials investigated pembrolizumab plus chemotherapy versus chemotherapy and two trials investigated pembrolizumab alone versus chemotherapy. All the trials used the 22C3 pharmDx assay (Agilent Technologies) to assess PD-L1 expression with immunohistochemical method. All the included trials used standard-of-care chemotherapeutic regimens according to practice guidelines. The median follow-up time ranged from 7.8 months to 23.9 months. All the five trials provided ORR data; OS and PFS data were not reported in KEYNOTE-021 trial cohort G [4].

**Table 1:** Characteristics of Patients Comparing Pembrolizumab plus Chemotherapy or Pembrolizumab alone with Chemotherapy in Included Trials

Source	Histology	Therapeutic regimen	Chemotherapy Drug	No. of patients		NO. of response		PFS^a^(m)	HR for PFS	OS^a^(m)	HR for OS	Median Follow-up time (m)
				Pem/Pem + Chemo	Chemo	Pem/Pem + Chemo	Chemo					
KEYNOTE-021 2016, 2018	nonsquamous	Pem + Chemo vs. Chemo	AC 1) carboplatin (5 mg/ml/min Q3W) 2) pemetrexed (500 mg/m^2 Q3W)	20	17	16	6	NR	NR	NR	NR	23.9
KEYNOTE-189 2018	nonsquamous	Pem + Chemo vs. Chemo	AP or AC 1) cisplatin (75 mg/m^2 Q3W) or carboplatin (6 mg/ml/min Q3W) 2) pemetrexed (500 mg/m^2 Q3W)	132	70	81	16	NR	0.36 (0.25–0.52)	NR	0.42 (0.26–0.68)	10.5
KEYNOTE-407 2018	squamous	Pem + Chemo vs. Chemo	PC 1) carboplatin (6 mg/ml/min Q3W) 2) paclitaxel(200 mg/m^2 Q3W) or nab-paclitaxel (100 mg/m^2 Q1W)	73	73	44	24	8.0 vs. 4.2	0.37 (0.24–0.58)	NR	0.64 (0.37–1.10)	7.8
KEYNOTE-024 2016, 2017	suqamous and nonsquamous	Pem vs. Chemo	AP or AC or PC or GP or GC 1) cisplatin (75 mg/m^2 Q3W) or carboplatin (5-6 mg/ml/min Q3W) 2) pemetrexed (500 mg/m^2 Q3W) or paclitaxel (200 mg/m^2 Q3W) or Gemicitabine (1250 mg/m2 d1,8 of Q3W)	154	151	70	45	10.3 vs. 6.0	0.50 (0.37–0.68)	30.0 vs. 14.2	0.63 (0.47–0.86)	25.2
KEYNOTE-042 2018	suqamous and nonsquamous	Pem vs. Chemo	AC or PC 1) carboplatin (5-6 mg/ml/min Q3W) 2) pemetrexed (500 mg/m^2 Q3W) or paclitaxel (200 mg/m^2 Q3W)	299	300	118	96	7.1 vs. 6.4	0.81 (0.67–0.99)	20.0 vs. 12.2	0.69 (0.56–0.85)	12.8

^a^Data presented as “Pem/Pem + Chemo vs. Chemo”

Abbreviation: *Pem* Pembrolizumab, *Chemo* Chemotherapy, *NR* Not Reported, *HR* Hazard Ratio, *PFS* Progression-free Survival, *OS* Overall survival;

### Direct meta-analysis

**Fig. 1:**
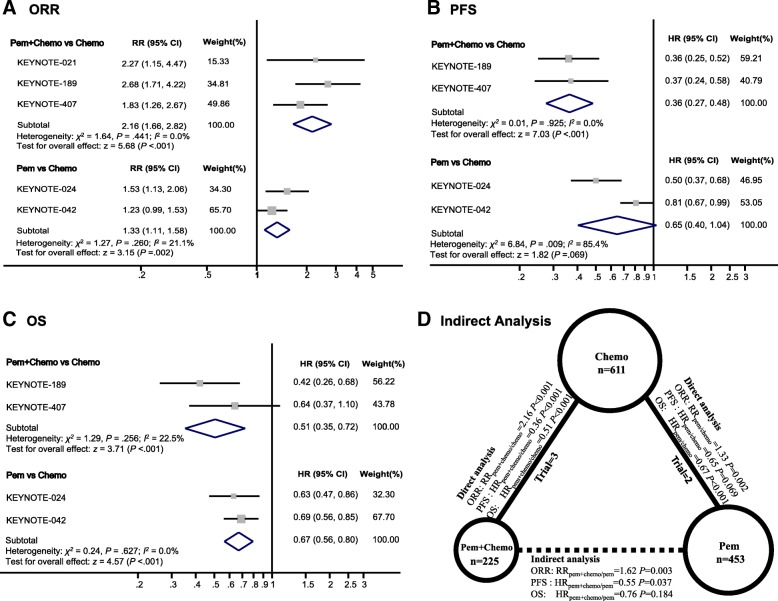
Direct Comparisons between Pembrolizumab plus Chemotherapy or Pembrolizumab Alone with Chemotherapy and Indirect Comparison between Pembrolizumab plus Chemotherapy versus Pembrolizumab Alone. **a**, **b** and **c** showed the Forest plot of risk ratios (RRs) and hazard ratios (HRs) directly comparing objective response rate (**a**), progression-free survival (**b**), and overall survival (**c**) between pembrolizumab plus chemotherapy or pembrolizumab alone with chemotherapy. The size of the data markers (squares) corresponds to the weight of the study in the meta-analysis. The horizontal line crossing the square represents the 95% CI. The diamonds represent the estimated overall effect, based on the meta-analysis. In **d**, solid lines represented the existence of direct comparisons between treatment regimens, and dashed line represented the indirect comparison between pem + chemo versus pem. The size of the circle corresponds to the enrolled patient number. All statistical tests were 2-sided. Abbreviations: *Pem* Pembrolizumab, *Chemo* Chemotherapy

Significant difference of ORR was observed in favor of pembrolizumab plus chemotherapy versus chemotherapy (RR_pem + chemo/chemo_ 2.16, 95% CI 1.66–2.82; *P* < 0.001; heterogeneity, *P* = 0.441). And for pembrolizumab vs chemotherapy, the pooled RR_pem/chemo_ was 1.33 (95% CI 1.11–1.58; *P* = 0.002; heterogeneity, *P* = 0.260) (Fig. 1a).

For PFS, pembrolizumab plus chemotherapy significantly reduced the risk of disease progression compared with chemotherapy (HR_pem + chemo/chemo_, 0.36; 95% CI 0.27–0.48; z = 7.03, *P* < 0.001; heterogeneity, *P* = 0.925). While pembrolizumab monotherapy failed to demonstrate significant improvement in PFS (HR_pem/chemo_, 0.65; 95% CI 0.40–1.04; z = 1.82, *P* = 0.069; heterogeneity, *P* = 0.009) (Fig. 1b).

In terms of OS, both pembrolizumab plus chemotherapy (HR_pem + chemo/chemo_, 0.51; 95% CI 0.35–0.72; z = 3.71, *P* < 0.001) and pembrolizumab monotherapy (HR_pem/chemo_, 0.67; 95% CI 0.56–0.80; z = 4.57, *P* < 0.001) significantly decreased the risk of death compared with chemotherapy (Fig. 1c).

### Indirect meta-analysis

Figure 1d showed the relationship of the indirect comparisons. The results indicated that patients treated with pembrolizumab plus chemotherapy had better clinical outcomes including ORR (RR_pem + chemo/pem_ 1.62, 95% CI 1.18–2.23; *P* = 0.003) and PFS (HR_pem + chemo/pem_ 0.55, 95% CI 0.32–0.97; *P* = 0.037) than those treated with pembrolizumab alone. However, there was only a trend towards improved OS with the three-drug combination therapy (HR_pem + chemo/pem_ 0.76, 95% CI 0.51–1.14; *P* = 0.184).

## Discussion

In this hypothesis-generating meta-analysis, we found that pembrolizumab plus chemotherapy is superior to pembrolizumab alone for first-line treatment of patients with advanced NSCLC and a PD-L1 TPS of ≥50%, in terms of ORR and PFS. A trend towards improved OS is also observed in the three-drug combination group.

PD-L1 is an established biomarker for selecting patients for first-line treatment with pembrolizumab monotherapy [1]. Although it may be tempting to believe that pembrolizumab monotherapy attains a better toxicity profile while retaining survival benefit in patients with a PD-L1 TPS of at least 50%. The challenge is that less than 50% of patients with advanced NSCLC ever receive second-line therapy due to rapid deterioration during disease progression [8]. Therefore, maximizing the chance of response to first-line treatment and delaying the occurrence of drug resistance is clinically relevant. Another challenge is the intratumoral heterogeneity of PD-L1 expression [9]. A fine-needle aspiration specimen does not represent the whole picture of the tumour and high PD-L1 expression detected in this circumstance might be “false positive”. Additionally, the cutoff value of 50% is not ideal for benefit stratification. A retrospective study found that pembrolizumab only produced moderate efficacy in patients with a PD-L1 TPS of 50–74% (ORR 21.6%; PFS 3.2 months; OS 20.6 months) or 50–89% (ORR 25.2%; PFS 3.7 months; OS 15.2 months) [10], indicating that the exact beneficial population might be those with even higher PD-L1 level, though the optimal cutoff remains not illustrated. These challenges probably explained the phenomenon that pembrolizumab monotherapy only produces a response rate of 40–45% and that the separation of survival curves is in a delayed manner [5, 7].

Our pooled analysis indicates that pembrolizumab monotherapy did not significantly improved PFS compared with chemotherapy while pembrolizumab plus chemotherapy outperforms chemotherapy in terms of all the tested outcomes including ORR, PFS and OS. Indirect comparison shows that the addition of chemotherapy to pembrolizumab further increases the chance of response by 62%. Additionally, the risk of disease progression and death is reduced by 45 and 24%, respectively. Although the improvement of OS with the three-drug combination versus pembrolizumab single agent is not statistically significant, it is likely due to the short duration of follow-up in KEYNOTE-407 trial [6]. An update analysis with extended follow-up will be needed. Our findings lend support for the hypothesis that chemotherapeutic agents may exert immune-potentiating effects under certain circumstance. Based on these data, it may be reasonable to recommend that patients with high tumor volume to be treated with the combinatorial therapy to produce deeper and longer response, while patients with low tumor volume or with very high PD-L1 TPS to be treated with pembrolizumab alone.

A strength of this work is the quality of evidence available and used in the meta-analysis. Source data were obtained from five well-designed randomised controlled trials involving over 1000 patients. The experimental drug and methods for PD-L1 expression is the same. Thus, the meta-analysis could overcome the problem of inadequate power of each individual trial by pooling data together and minimize between-study heterogeneity. Albeit the strength above, we encountered several limitations during this study. First of all, our meta-analysis relies on published results rather than on individual patients’ data. Secondly, we lacked data from head-to-head comparison. Finally, the data from pembrolizumab plus chemotherapy are retrieved from subgroup analyses. Therefore, the interpretation of the results needs additional caution. However, there was no important difference between trials with pembrolizumab plus chemotherapy and trials with pembrolizumab monotherapy included for the analyses, which makes the indirect comparison reliable to some extent. Given these limitations, head-to-head randomized trials will be required to directly compare pembrolizumab plus chemotherapy against pembrolizumab alone. Future researches should also explore the optimal cutoff value of PD-L1 above which pembrolizumab is non-inferior to pembrolizumab plus chemotherapy.

In conclusion, the addition of chemotherapy to pembrolizumab as first-line treatment further improves the outcomes of patients with advanced NSCLC and a PD-L1 TPS of at least 50%. With proved survival benefit, manageable toxicities and avoidance of PD-L1-based patient selection, clinicians could prefer pembrolizumab plus chemotherapy in patients without contraindications, especially for those with high tumor burden.

## Additional file

Additional file 1: **Supplemental Methods.** Search strategies and number of studies yielded from each database. **Table S1.** Quality assessment: risk of bias by Cochrane Collaboration’s tool. **Figure S1.** Trial Selection Process (PDF 337 kb)

## Abbreviations

AACR: American Association for Cancer Research
advanced NSCLC: Advanced non-small cell lung carcinoma
ASCO: American Society of Clinical Oncology
Chemo: Chemotherapy
CI: Confidence interval
ESMO: European Society of Medical Oncology
HR: Hazard ratio
ORR: Objective response rate
OS: Overall survival
PD-1: Programmed cell death 1
PD-L1: Programmed cell death-ligand 1
Pem: Pembrolizumab
PFS: Progression-free survival
RR: Relative risk
SE: Standard error
TPS: Tumor proportion score tumor proportion score
WCLC: World Conference on Lung Cancer
