# Supplementary material for: First-line treatment for patients with advanced non-small cell lung carcinoma and high PD-L1 expression: pembrolizumab or pembrolizumab plus chemotherapy
Source: J Immunother Cancer. 2019 May 3;7:120. doi: 10.1186/s40425-019-0600-6 (PMC6500047; doi:10.1186/s40425-019-0600-6)

Supplemental Methods

Search strategies and number of studies yielded from each database.

Pubmed: 75 Results

(((("Carcinoma, Non-Small-Cell Lung"[Mesh]) OR ((((NSCLC[Title/Abstract] OR "Non Small Cell"[Title/Abstract] OR "Non-Small-Cell"[Title/Abstract] OR "Non-Small Cell"[Title/Abstract] OR "Non-Small Cell"[Title/Abstract]))) AND lung[Title/Abstract]))) AND (("pembrolizumab" [Supplementary Concept] OR "lambrolizumab"[Title/Abstract] OR "Keytruda"[Title/Abstract] OR "MK-3475"[Title/Abstract]))) AND ((("clinical trials as topic"[MeSH Terms] OR "trial"[Title/Abstract] OR "study"[Title/Abstract])))

EMbase: 106 Results

('pembrolizumab'/exp OR 'lambrolizumab':ab,ti OR 'Keytruda':ab,ti OR 'MK-3475':ab,ti) AND (('non small cell lung cancer'/exp OR ('lung':ab,ti AND ('NSCLC':ab,ti OR 'Non Small Cell':ab,ti OR 'Non-Small-Cell':ab,ti OR 'Non-Small Cell':ab,ti OR 'Non-Small Cell':ab,ti))) AND ('randomized controlled trial'/exp))

Cochrane: 162 results, 158 trials

#1 MeSH descriptor: [Carcinoma, Non-Small-Cell Lung] explode all trees

#2 “lung” AND ("Non Small Cell" OR "Non-Small Cell" OR "Non-Small-Cell")

#3 "pembrolizumab" or "lambrolizumab" or "Keytruda" or "MK-3475"

4 (#1 OR #2) AND #3

Supplemental Table 1. Quality assessment: risk of bias by Cochrane Collaboration's tool

Trial	Sequence generation	Allocation Concealment	Bliding	Incomplete outcome data	Selective reporting	Other source of bias
KEYNOTE-021 2016, 2018	Adequate	Adequate (Central allocation)	Adequate (Independent Radiologic review)	Adequate	Inadequate (PFS、OS was not reported)	
KEYNOTE-189 2018	Adequate	Adequate (Central allocation)	Adequate (Independent Radiologic review)	Adequate	Adequate	
KEYNOTE-407 2018	Adequate	Adequate (Central allocation)	Adequate (Independent Radiologic review)	Adequate	Adequate	
KEYNOTE-024 2016, 2017	Adequate	Adequate (Central allocation)	Adequate (Independent Radiologic review)	Adequate	Adequate	
KEYNOTE-042 2018	Adequate	Adequate (Central allocation)	Adequate (Independent Radiologic review)	Adequate	Adequate	Data from the abstract and the presentation slides

Supplemental Figure 1. Trial Selection Process

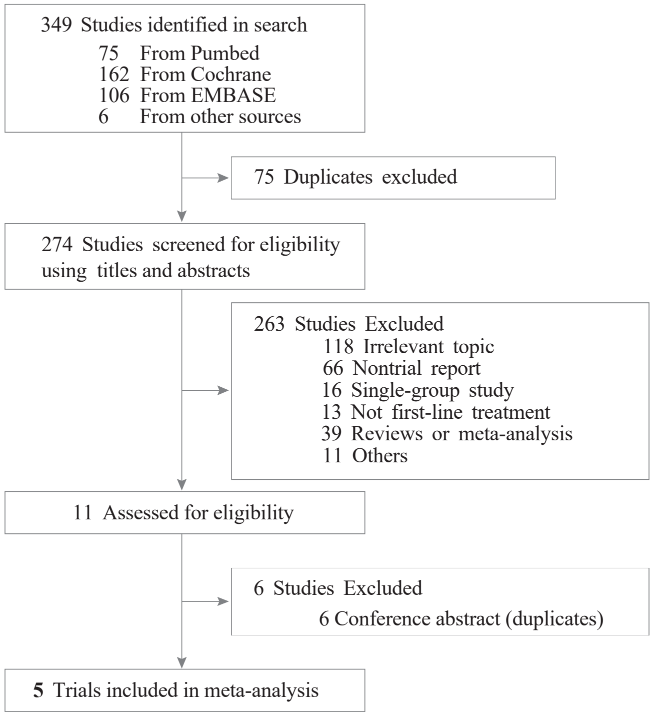

Supplement: Supplementary file 1 — Supplemental Methods. Search strategies and number of studies yielded from each database. Table S1. Quality assessment: risk of bias by Cochrane Collaboration’s tool. Figure S1. Trial Selection Process (PDF 337 kb) [file 40425_2019_600_MOESM1_ESM.pdf]
